# Associations between health information source and childhood vaccination

**DOI:** 10.3389/fpubh.2025.1627916

**Published:** 2025-09-29

**Authors:** Karly S. Geller, Skyler J. Montaine-O'Brien, Paul W. Branscum, Brandy N. Reeves-Doyle

**Affiliations:** ^1^Department of Kinesiology, Nutrition, and Health, Miami University, Oxford, OH, United States; ^2^College of Public Health, Ohio State University, Columbus, OH, United States

**Keywords:** pediatric immunization, vaccine misinformation, parental vaccine concerns, unverified sources, verified health information

## Abstract

The credibility of health-related information sources may influence parental decisions regarding childhood vaccinations. This study examined whether the type of health information source used by parents (verified vs. unverified) was associated with their child's vaccination outcomes. A national sample of 887 parents (55.1% male; mean age = 36.42 years, standard deviation = 12.29) completed an anonymous online survey. Participants reported demographic characteristics, primary health information sources, and whether their child received 12 vaccine recommendations by the Center of Disease Control and Prevention (CDC). Health information sources were categorized as primarily from verified (healthcare providers, scientific journals) or unverified (family/friends, social media, opinion blogs). Compared to those using unverified sources, verified source users had significantly higher odds of vaccinating their child across most vaccines, including DTaP, Hib, Hepatitis A, Hepatitis B, influenza, MMR, MCV4, pneumonia, IPV, and RV (all *p*s < 0.01). Verified source users also reported significantly higher overall vaccination rates (*p* < 0.001). These associations remained significant after adjusting for key demographic covariates (e.g., age, household size, number of children). No significant differences were found for the chickenpox or COVID-19 vaccines. Results underscore the importance of health information credibility in promoting vaccine adherence and suggest that targeted efforts to improve access to verified health information may help address childhood vaccination gaps.

## Introduction

Childhood vaccination remains one of the most effective public health interventions, preventing thousands of infectious disease cases annually in the United States, such as measles, mumps, tetanus, and diphtheria (1). Worldwide it is also estimated that approximately four million deaths are prevented annually due to childhood vaccinations (Centers for Disease Control and Prevention [CDC] (2). Vaccine hesitancy among parents, however, has increased in recent years, despite the widespread availability of vaccines, fueled in part by the proliferation of online health misinformation (3, 4). Although healthcare providers continue to serve as trusted sources of vaccine information, many parents seek supplemental information from social media, blogs, and other unverified sources, where misinformation can spread rapidly (5, 6).

Exposure to vaccine misinformation has been linked to lower vaccination intentions and reduced vaccine uptake among both adults and children (4, 6). Studies show that misinformation often exploits emotional appeals, undermines trust in scientific institutions, and disproportionately affects individuals with lower health literacy (7, 8). Health literacy, including vaccine literacy, is a key determinant of vaccination behavior (9). Individuals with higher literacy are more likely to seek verified health information, which in turn promotes vaccine confidence, reduces susceptibility to misinformation, and increases uptake (9, 10). Misinformation regarding routine childhood immunizations, such as the measles, mumps and rubella (MMR) vaccine, has contributed to localized outbreaks of preventable diseases such as measles (11). Parents who rely primarily on non-credible or unverified sources may be more likely to delay or refuse vaccinations, whereas those who use verified health sources, such as healthcare providers or public health agencies, demonstrate higher rates of adherence (5, 12, 13).

Parental characteristics such as age, race, education, and income can also influence the types of health information sources used when making vaccine decisions. Certain demographic groups may be more vulnerable to misinformation due to greater exposure to unregulated online content or skepticism toward traditional medical institutions (14, 15). Research indicates that younger, more educated parents are more likely to seek vaccine information from unverified online sources (16). Racial and ethnic disparities in access to accurate health information have also been documented (17), and individuals with lower incomes often report lower confidence in evaluating the credibility of health information (18). Parents with one child are more likely to use unverified vaccine information sources, though evidence on household size is limited (19). Direct comparisons by sexual orientation are scarce, but sexual minority populations may face distinct barriers to vaccine information and uptake (20). Political identity also influences information use. Democrats more often rely on verified sources and show higher vaccine confidence, while Republicans more often rely on unverified sources and Independents fall in between (21). These trends underscore the need to examine how demographic factors shape information-seeking behaviors and vaccine uptake. Tailoring communication strategies to account for these differences may enhance vaccine confidence and improve coverage among at-risk groups (22, 23).

Given the ongoing challenges presented by health misinformation, it is important to better understand how sources of information parents use relate to their child's vaccination outcomes. This study examined whether parents who use verified vs. unverified health information sources differ in demographic characteristics and the likelihood of vaccinating their child for recommended vaccines. Differences in the use of unverified or verified health information between demographic subgroups was expected to mirror previous research, suggesting higher likelihood of using verified information among older parents with lower education levels. It was also hypothesized that parents who relied on verified health information sources would report higher rates of child vaccination compared to those who used unverified sources.

## Method

Eligible parents/legal guardians with at least one child under age 18 were asked whether their child had received 12 recommended childhood vaccinations, 11 of which are typically administered within the first 23 months after birth: Varicella (chickenpox); COVID-19; Diphtheria, Tetanus, and acellular Pertussis (DTaP); Haemophilus influenzae type b (Hib); Hepatitis A; Hepatitis B; Influenza (flu); Measles, Mumps, and Rubella (MMR); Pneumococcal conjugate vaccine (pneumonia); Inactivated Poliovirus Vaccine (IPV); and Rotavirus vaccine (RV). Meningococcal conjugate vaccine (MCV4), typically given at 11 years of age, was also included (22). Although the HPV vaccine is recommended during adolescence, it was excluded from this study due to its later administration and the reduced reliability of parental recall (24, 25). Additionally, because data collection occurred in 2022 prior to the CDC's recent recommendations for respiratory syncytial virus (RSV) immunization, uptake of the RSV vaccine was not assessed. The survey was administered through Qualtrics and distributed across multiple social media platforms, including Facebook, LinkedIn, Instagram, Twitter, and Snapchat. This social media recruitment resulted in a convenience sample. The study was approved by the Institutional Review Board of Miami University (Oxford, Ohio; Protocol #02162r), and informed consent was obtained from all participants prior to data collection. Participants accessed the survey via a social media link and gave informed consent by selecting ‘Yes' before beginning. Surveys with missing or incomplete vaccination data were excluded from the final analysis.

### Measurement

Participants self-reported their demographic characteristics, including age, sex, ethnicity, sexual orientation, highest education level, household income, political party affiliation, religion, household size, and number of children. Participants also indicated their primary source of health-related information (“*Where do you primarily get your medical information from?*), categorized as verified (healthcare providers and/or scientific journals) or unverified (family/friends, social media, and/or opinion blogs). Child vaccination status was assessed by asking participants to check “yes” (vaccinated) or “no” (not vaccinated) for each of the aforementioned vaccines. One example question was, “*Has your child been vaccinated against chickenpox*.” A total vaccination score was calculated by summing the number of vaccines received, with possible scores ranging from 0 to 12.

### Statistical analysis

All analyses were conducted using IBM SPSS Statistics, Version 29, with statistical significance at *p* < 0.05 (two-tailed). Descriptive statistics (means, standard deviations [SD]), and frequencies) were used to characterize the sample. Independent samples *t*-tests and chi-square tests were conducted to compare demographic characteristics between verified and unverified information source groups. Potential covariates were tested for each vaccine outcome, including age, household size, number of children, sex, ethnicity, education, household income, sexual orientation, and political affiliation. Covariates significantly associated with each outcome were retained in adjusted models. Separate multivariable logistic regression models were conducted for each vaccine with a significant unadjusted group difference, estimating adjusted odds ratios (aORs), 95% confidence intervals (CIs), and *p*-values. Total vaccination scores were compared between groups using an independent samples *t*-test. An a priori sample size calculation of 285 participants was determined using G Power, using the following parameters: (logistic regression) alpha (0.05); power (1-beta) 0.80; and an expected odds ratio of 4.62, which was calculated based on a previous national sample that showed on average 58% of children are up to date on normally scheduled vaccines, while 23% follow an alternate or unknown pattern (26).

## Results

The sample included 887 participants (55.1% male, 44.9% female) with a mean age of 36.42 years (SD = 12.29). Table 1 summarizes participant demographics by types of health information sources. Verified source users had significantly less children than unverified information users (*p* < 0.001). Verified source users were more likely to identify as Hispanic/Latino, report higher household incomes, and identify as a Democrat (all *p*-values < 0.001). Verified information users were also more likely to have a bachelor's degree (*p* = 0.002) and to identify as heterosexual (*p* = 0.001). No significant differences were observed between groups for age, household size, or sex.

**Table 1:** Participant demographics by types of health information sources (verified vs. unverified).

**Characteristic**	**Total sample (*N* = 887)**	**Verified source users (*n* = 365)**	**Unverified source users (*n* = 522)**	**Test statistic (t or χ^2^)**	***p*-value**
Age, M (SD) US (2023) Median: 38.9 (27)	36.42 (12.29)	35.90 (13.12)	36.80 (11.64)	−1.05	0.30
Household size, M (SD) US (2023): 3.09 (0.01) (27)	3.19 (0.98)	3.15 (0.91)	3.23 (1.02)	−1.24	0.22
Number of children, M (SD) US TFR (2024): 1.62 (28)	1.44 (0.62)	1.34 (0.64)	1.50 (0.60)	**–3.67**	**< 0.001**
Number of vaccinations (0–12), M (SD)	9.79 (3.18)	10.63 (2.22)	9.20 (3.59)	**7.31**	**< 0.001**
Sex (%)				0.72	0.40
Female US (2023): 50.6% (29)	44.9	39.7	60.3		
Male US (2023): 49.4% (29)	55.1	42.5	57.5		
Ethnicity (%)				**28.11**	**< 0.001**
White, non-hispanic US (2023): 58.5% (27)	59.8	40.6	59.4		
Hispanic/Latino US (2023): 19.3% (27)	13.1	60.3	39.7		
African American US (2023): 13.5% (27)	10.5	26.9	73.1		
Native American US (2020): 0.9% (29)	9.6	35.3	64.7		
Asian US (2023): 6.5% (27)	4.1	33.3	66.7		
Other	2.8	48.0	52.0		
Education (%)				**14.51**	**0.002**
High school or less US high school (2023): 25.9% (27)	10.4	43.4	56.5		
Some college/associate/certificate US (2023): 27.7% (27)	43.2	35.7	64.3		
Bachelor's degree US (2023): 21.8% (27)	34.2	49.5	50.5		
Graduate degree US (2023): 14.3% (27)	12.1	36.4	63.5		
Household income (%) US Median (2023) $77,719 (27)				**71.84**	**< 0.001**
< $40,000	17.9	47.1	52.9		
$40,000–$79,999	45.2	25.7	74.3		
≥$80,000	36.9	56.2	43.8		
Sexual orientation (%)				**13.60**	**0.001**
Heterosexual	89.4	42.7	57.3		
LGBQ US (2016–2017): 5–7% (30)	10.6	29.0	71.0		
Political affiliation (%)				**36.36**	**< 0.001**
Democrat US (2020): 37% (31)	34.0	54.7	45.3		
Republican US (2020): 32% (31)	24.2	30.1	69.8		
Independent/Other US (2020): 31% (31)	41.8	63.5	36.5		

χ^2^, chi-square; M, mean; SD, standard deviation; TFR, Total Fertility Rate; LGBQ, lesbian, gay, bi-sexual, Queer. Statistically significant values (*p* < 0.05) are bolded.

Table 2 summarizes the associations between types of health information sources and child vaccination status across all vaccines. No significant associations were found for the chickenpox or COVID-19 vaccines. Use of verified health information sources was significantly associated with higher odds of child vaccination for all other vaccines, including DTaP [OR = 4.52, 95% CI (2.94, 6.96)], Hib [OR = 2.89, 95% CI (1.95, 4.29)], Hepatitis A [OR = 3.78, 95% CI (2.53, 5.65)], Hepatitis B [OR=3.34, 95% CI (2.18, 5.11)], Flu [OR = 1.81, 95% CI (1.23, 2.66)], MMR [OR = 4.19, 95% CI (2.66, 6.59)], MCV4 [OR = 2.61, 95% CI (1.82, 3.75)], pneumonia [OR = 2.36, 95% CI (1.65, 3.39)], IPV [OR = 2.48, 95% CI (1.69, 3.65)], and RV [OR = 2.09, 95% CI (1.48, 2.96)] (all *p* < 0.01).

**Table 2:** Chi-square associations between types of health information sources and child vaccination status across vaccines.

**Vaccine**	**χ^2^**	***p*-value**	**OR**	**95% CI**
Chickenpox	0.13	0.72	1.11	0.63, 1.95
COVID-19	0.46	0.50	0.87	0.58, 1.30
DTaP	53.13	< 0.001	4.52	2.94, 6.96
Hib	29.55	< 0.001	2.89	1.95, 4.29
Hepatitis A	46.14	< 0.001	3.78	2.53, 5.65
Hepatitis B	33.32	< 0.001	3.34	2.18, 5.11
Influenza	9.19	0.002	1.81	1.23, 2.66
MMR	42.92	< 0.001	4.19	2.66, 6.59
MCV4	28.24	< 0.001	2.61	1.82, 3.75
Pneumonia	22.55	< 0.001	2.36	1.65, 3.39
IPV	22.22	< 0.001	2.48	1.69, 3.65
RV	17.89	< 0.001	2.09	1.48, 2.96

χ^2^, chi-square (degrees of freedom=1); OR, Odds Ratio; CI, Confidence Interval; DTaP, Diphtheria, Tetanus, and acellular Pertussis vaccine; Hib, Haemophilus influenzae type b vaccine; MMR, Measles, Mumps, and Rubella vaccine; MCV4, Meningococcal conjugate vaccine; IPV, Inactivated Poliovirus Vaccine; RV, Rotavirus vaccine; Pneumonia, Pneumococcal conjugate vaccine; Flu, Influenza vaccine; Chickenpox, Varicella vaccine; COVID-19, Coronavirus Disease 2019 vaccine; Hepatitis A, Hepatitis A vaccine; Hepatitis B, Hepatitis B vaccine.

Table 3 presents association between types of health information sources and child vaccination status. After controlling for significant covariates, verified information source use remained significantly associated with higher odds of child vaccination across all models. Participants using verified sources had 4.2 times greater odds of DTaP vaccination [aOR = 4.18, 95% CI (2.66, 6.55), *p* < 0.001] and 2.5 times greater odds of Hib vaccination [aOR=2.49, 95% CI (1.64, 3.77), *p* < 0.001] compared to those using unverified sources. Similarly, verified source use was associated with higher odds of Hepatitis A [aOR = 3.33, 95% CI (2.19, 5.06), *p* < 0.001], Hepatitis B [aOR = 2.85, 95% CI (1.83, 4.45), *p* < 0.001], and influenza vaccination [aOR = 1.53, 95% CI (1.03, 2.28), *p* = 0.037]. Participants using verified sources also had greater odds of vaccinating for MMR [aOR = 3.88, 95% CI (2.45, 6.14), *p* < 0.001], MCV4 [aOR = 2.40, 95% CI (1.66, 3.47), *p* < 0.001], pneumonia [aOR = 2.01, 95% CI (1.38, 2.91), *p* < 0.001], IPV [aOR = 2.24, 95% CI (1.51, 3.31), *p* < 0.001], and RV [aOR = 1.76, 95% CI (1.23, 2.51), *p* = 0.002].

**Table 3:** Adjusted odds ratios for the association between types of health information sources and child vaccination status, controlling for significant covariates.

**Vaccine**	**aOR**	**95% CI**	***p*-value**
DTap	4.18	2.66, 6.55	< 0.001
Hib	2.49	1.64, 3.77	< 0.001
Hepatitis A	3.33	2.19, 5.06	< 0.001
Hepatitis B	2.85	1.83, 4.45	< 0.001
Flu	1.53	1.03, 2.28	0.037
MMR	3.88	2.45, 6.14	< 0.001
MCV4	2.40	1.66, 3.47	< 0.001
Pneumonia	2.01	1.38, 2.91	< 0.001
IPV	2.24	1.51, 3.31	< 0.001
RV	1.76	1.23, 2.51	0.002

aOR, adjusted Odds Ratio; CI, Confidence Interval; DTaP, Diphtheria, Tetanus, and acellular Pertussis vaccine; Hib, Haemophilus influenzae type b vaccine; MMR, Measles, Mumps, and Rubella vaccine; MCV4, Meningococcal conjugate vaccine; IPV, Inactivated Poliovirus Vaccine; RV, Rotavirus vaccine; Pneumonia, Pneumococcal conjugate vaccine; Flu, Influenza vaccine; Chickenpox, Varicella vaccine; COVID-19, Coronavirus Disease 2019 vaccine; Hepatitis A, Hepatitis A vaccine; Hepatitis B, Hepatitis B vaccine. Adjusted for covariates significant in bivariate screening. Covariates included were: DTap (sex, education level, religion, and number of children), Hib (sexual orientation, religion, and number of children), Hepatitis A (sexual orientation, religion, and household number), Hepatitis B (religion, marital status, and number of children), Flu (religion and number of children), MMR (political affiliation, religion), MCV4 (religion), pneumonia (religion, household number), IPV (religion), and RV (religion and household number).

## Discussion

The current study demonstrated that parents who relied on verified health information sources were significantly more likely to vaccinate their children for nearly all recommended vaccines compared to those using unverified sources. These findings align with previous research highlighting the critical role that information source credibility plays in shaping vaccination behaviors (4, 6). Importantly, this underscores how vaccine health literacy supports trust in credible sources and enables parents to make informed immunization decisions (9, 10). Parents using verified sources had substantially higher odds of vaccinating across vaccines such as DTaP, Hib, MMR, and Hepatitis B, suggesting that access to accurate information continues to be a central determinant of vaccine adherence in the United States. The significant association with total vaccination completeness further supports that reliance on credible health information promotes broader compliance with vaccination schedules.

These results are consistent with studies demonstrating the damaging influence of misinformation on vaccination intent and uptake (5, 7). Misinformation, especially from social media and informal online networks, has been found to undermine trust in public health recommendations and exacerbate vaccine hesitancy (6, 32). Current findings build on this prior work by confirming that information source type not only influences intent but also translates into actual vaccination behaviors across multiple childhood vaccines. The absence of significant findings for COVID-19 and varicella vaccines may reflect broader societal factors, including polarized messaging around COVID-19 and perceived lower risk associated with chickenpox infection (33, 34).

Demographic differences between verified and unverified source users were also notable. Parents who used verified sources were more likely to have higher education levels, higher household income, and Democratic political affiliation, patterns that mirror national data linking sociodemographic factors with health information-seeking behaviors and vaccine acceptance (13, 21, 35). In this sample, verified-source users were more often Hispanic/Latino. This aligns with data showing that Hispanic adults often seek out verified health information sources (36), though barriers like language and systemic mistrust persist (37). Contrary to prior research, parents with less children were more likely to rely on verified information (19). Additionally, heterosexual parents were more inclined to use verified health information compared to LGBQ parents; further research is needed to explore the diverse subgroups within the LGBQ parenting community. These differences highlight the intersection of social determinants of health and information environments. Public health initiatives aiming to address vaccine hesitancy must account for these demographic patterns and focus outreach efforts on populations more likely to engage with less credible information sources.

Several strengths and limitations should be considered for this study. Strengths include the use of a large, diverse national sample and the inclusion of multiple child vaccination outcomes, allowing a comprehensive evaluation of associations across vaccines. However, the study relied on a convenience rather than a representative sample, which may limit the generalizability of findings. Limitations also include reliance on self-reported vaccination data, which may introduce recall bias, and the cross-sectional design, which precludes causal conclusions. Additionally, the categorization of verified vs. unverified sources was based on participant self-report, which may not fully capture the complexity of health information environments. Future research should incorporate longitudinal designs and objective measures of information exposure to better understand causal pathways.

## Data Availability

The raw data supporting the conclusions of this article will be made available by the authors, without undue reservation.
